# The Proprietary Medicine Question

**Published:** 1906-03

**Authors:** 


THE PROPRIETARY MEDICINE QUESTION.—*The Chicago Clinic*, etc., in discussing this matter says, that the sentiment which underlies the present efforts of certain medical men to protect the profession from imposition, and to make our therapy clean, reliable and trustworthy, is entirely laudable and commendable; but the extent to which some are permitting their enthusiasm to carry them is lamentable. The judgment passed upon many pharmaceutical preparations which have stood the test of time in the practices of thousands of successful medical men has seemed hasty and ill advised. It is questionable if it is right that a small faction of the American Medical Association should use the organ owned by all of its members to condemn or detract from the reputation of long established preparations—many of which are used regularly by a large number of the association. The average preparation which has been used by medical men of intelligence for years with good results must have something in its favor.

